# Mitochondrial Reactive Oxygen Species Formation Determines ACSL4/LPCAT2-Mediated Ferroptosis

**DOI:** 10.3390/antiox12081590

**Published:** 2023-08-09

**Authors:** Melanie Merkel, Bjarne Goebel, Moritz Boll, Aasha Adhikari, Viktoria Maurer, Dieter Steinhilber, Carsten Culmsee

**Affiliations:** 1 Institute of Pharmacology and Clinical Pharmacy, Philipps-University Marburg, Karl-von-Frisch-Str. 2, 35043 Marburg, Germany; melanie.merkel@pharmazie.uni-marburg.de; 2 Marburg Center of Mind, Brain, and Behavior—CMBB, Hans-Meerwein-Straße 6, 35032 Marburg, Germany; bollmo@students.uni-marburg.de (M.B.); adhikara@students.uni-marburg.de (A.A.); maurerv@students.uni-marburg.de (V.M.); 3 Institute of Pharmaceutical Chemistry, Goethe-University Frankfurt, Max-von-Laue-Str. 9, 60439 Frankfurt, Germany; goebel@pharmchem.uni-frankfurt.de (B.G.); steinhilber@em.uni-frankfurt.de (D.S.)

**Keywords:** mitochondrial ROS, ACSL4, LPCAT2, lipid peroxidation, ferroptosis, mitochondria, mitoquinone, RSL3, GPx4, HEK293T cells

## Abstract

Ferroptosis is a form of oxidative cell death that is characterized by enhanced lipid peroxidation and mitochondrial impairment. The enzymes acyl-CoA synthetase long-chain family member 4 (ACSL4) and lysophosphatidylcholine acyltransferase (LPCAT) play an essential role in the biosynthesis of polyunsaturated fatty acid (PUFA)-containing phospholipids, thereby providing the substrates for lipid peroxidation and promoting ferroptosis. To examine the impact of mitochondria in ACSL4/LPCAT2-driven ferroptosis, HEK293T cells overexpressing ACSL4 and LPCAT2 (OE) or empty vector controls (LV) were exposed to 1S, 3R-RSL3 (RSL3) for induction of ferroptosis. The ACSL4/LPCAT2 overexpression resulted in higher sensitivity against RSL3-induced cell death compared to LV-transfected controls. Moreover, mitochondrial parameters such as mitochondrial reactive oxygen species (ROS) formation, mitochondrial membrane potential, and mitochondrial respiration deteriorated in the OE cells, supporting the conclusion that mitochondria play a significant role in ACSL4/LPCAT2-driven ferroptosis. This was further confirmed through the protection of OE cells against RSL3-mediated cell death by the mitochondrial ROS scavenger mitoquinone (MitoQ), which exerted protection via antioxidative properties rather than through previously reported metabolic effects. Our findings implicate that mitochondrial ROS production and the accompanying organelle disintegration are essential for mediating oxidative cell death initiated through lipid peroxidation in ferroptosis.

## 1. Introduction

Ferroptosis is a form of iron-dependent regulated cell death (RCD) [1] that is distinct from other forms of programmed cell death, such as apoptosis, necrosis, and autophagy [2]. Ferroptosis is characterized by the accumulation of iron-dependent lipid peroxides, which ultimately leads to oxidative cell death [3]. Over the past years, ferroptosis has been implicated in a wide range of pathologies, including neurodegeneration, acute ischemic brain injury, and cardiac diseases [4,5].

One of the key features of ferroptosis is the disruption of lipid repair systems engaging glutathione (GSH), a key antioxidant that supports neutralizing of reactive oxygen species (ROS). This is mediated by the enzyme glutathione peroxidase 4 (GPx4), which converts reactive lipid hydroperoxides into harmless lipid alcohols. However, when the level of lipid peroxides exceeds the enzymatic capacity of GPx4, lipid ROS accumulate and cause oxidative damage to cellular components [6,7]. These ferroptosis mechanisms can be induced by erastin or glutamate through inhibition of the cystine/glutamate antiporter (system Xc^−^) [1]. Further, the antioxidative machinery of the cells can be targeted to induce ferroptosis by 1S, 3R-RSL3 (RSL3), which directly inhibits GPx4 [8,9].

Another important factor in ferroptosis is the presence of iron ions, which can react with lipid hydroperoxides to produce highly reactive free radicals that cause further damage to cell membranes and organelles. This iron-dependent lipid peroxidation is promoted by the enzyme acyl-CoA synthetase long-chain family member 4 (ACSL4), which is involved in the uptake and activation of polyunsaturated fatty acids (PUFAs), thereby providing the substrates, i.e., the fuel for detrimental lipid ROS formation during ferroptosis [10,11]. Thus, the biosynthesis of PUFA-containing phospholipids is a prerequisite for the initiation of ferroptosis [12], and it is accelerated in cells with high levels of plasma membrane phosphatidylethanolamines (PE) [11]. In particular, ACSL4 mediates the conversion of arachidonic acid (AA) to AA-CoA [10,13,14], and AA-CoA is further esterified into AA-PE by lysophosphatidylcholine acyltransferase 3 (LPCAT3) [15,16]. The formation of lipid ROS is catalyzed via lipoxygenases (LOX), e.g., 5-LOX or 12/15-LOX [17], which both mediate the oxidation of free AA or by 12/15-LOX, which catalyzes the oxidation of AA-PE to form AA-OOH-PE [18,19].

Mitochondria may play a critical role in ferroptosis pathways, e.g., by regulating iron metabolism and storage, lipid peroxidation, glutathione levels, and energy metabolism. In addition, mitochondria are responsible for maintaining redox balance and generating energy through oxidative phosphorylation (OXPHOS), which in turn is a constant source of ROS production. Substantial mitochondrial dysfunction and damage include loss of mitochondrial membrane potential, mitochondrial ROS accumulation, and impaired respiration, which is followed by mitochondrial outer membrane permeabilization (MOMP) and the detrimental release of cytochrome c or apoptosis-inducing factor (AIF) and, therefore, has been referred to as the point of no return after initiation of (oxidative) cell death in different cell types [8,20,21,22,23,24].

Whether ferroptosis initiated through ACSL4 and LPCAT2 requires mitochondrial mechanisms [25,26] has not been fully clarified yet and is, therefore, our point of interest. It has been suggested that the generation of lipid-based ROS is a major hallmark of ferroptosis, and mitochondria are the main intracellular source [2,26,27,28]. In addition, reduced GSH and GPx4 inactivation have been linked to lipid peroxidation derived from 12/15-LOX, which results in the translocation of AIF from the mitochondria to the nucleus [8,17,25,29].

This led us to the question of addressing the involvement of mitochondria in ACSL4/LPCAT2-driven ferroptosis via ROS production and whether it can be intercepted by mitochondria-targeted antioxidants or through metabolic interventions at the level of mitochondria. To obtain further insights into the role of mitochondria in ferroptosis, we investigated whether overexpression of ACSL4 and LPCAT2 (OE) in HEK293T cells affected mitochondrial mechanisms of oxidative cell death in a model of RSL3-mediated ferroptosis and evaluated strategies of mitochondria-targeted protection. The results obtained in this study demonstrate an increased susceptibility against ferroptosis and involvement of mitochondria in RSL3-induced ferroptosis in ACSL4/LPCAT2 OE cells compared to empty vector (LV) control cells. Ferroptosis in HEK293T OE cells was prevented by the mitochondrial ROS scavenger mitoquinone (MitoQ) but not by metabolic inhibition of mitochondrial OXPHOS.

## 2. Materials and Methods

### 2.1. Cell Culture, Compounds, and Treatments

HEK293T cells are immortalized human embryonic kidney cells that express a mutant version of the temperature-sensitive SV-40 large T antigen. HEK293T cells were stably transfected by the sleeping beauty system [30] for overexpression of the proteins ACSL4 and LPCAT2 (OE) and empty vector control (LV), with green fluorescent protein (GFP) transfection as control.

The cells were cultured in Dulbecco’s modified Eagle’s medium (DMEM High Glucose; Capricorn Scientific GmbH, Ebsdorfergrund, Germany), which was supplemented with 10% fetal calf serum superior (Merck KGaA, Darmstadt, Germany), 1% penicillin, streptomycin (Capricorn, Ebsdorfergrund, Germany), and 2% L-Alanyl-L-glutamine (Capricorn, Ebsdorfergrund, Germany), and regularly checked for mycoplasma contamination.

1S,3R-RSL3 (1 mM in DMSO), short RSL3, was synthesized as specified before [8]. A total of 24–30 h in advance of each experiment, cells were seeded at the following densities and cultured at 37 °C and 5% CO_2_ in a Heracell 150 CO_2_-incubator (Thermo Fisher Scientific, Darmstadt, Germany): 7000–11,000 cells per well in 96-well plates, 96-well xCELLigence E-plates (Roche, Applied Science, Penzberg, Germany), or XFe96-well microplates (Agilent Technologies, Waldbronn, Germany); 43,000–60,000 cells per well in 24-well plates; and 300,000–450,000 cells per well in 6-well plates, respectively.

Ferroptotic cell death was induced 24–30 h after cell seeding by 0.01–1 µM RSL3 for the indicated times, depending on the experimental design. The different compounds were either co-treated (added at the same time) or pre-treated (applied in advance to RSL3 for the indicated time) with RSL3. To initiate cell death, 1 mM of the stock solution was thawed and then further diluted with DMEM to the required concentrations. After treatment, the cells were incubated at 37 °C and analyzed or harvested after the indicated time point.

Ferrostatin-1 (Fer-1) (Sigma-Aldrich, Taufkirchen, Germany) is one of the earliest and most widely studied ferroptosis inhibitors [1]. It acts as a potent lipid peroxidation inhibitor and functions by directly scavenging lipid peroxides in the cell membrane [31]. The inhibitor was dissolved in DMSO to a stock concentration of 10 mM and stored at −20 °C. The concentrations used ranged between 0.1 and 15 µM.

Two different 5-LOX inhibitors were used to inhibit cell death induced by RSL3. Zileuton (Cayman Chemical, Ann Arbor, MI, USA) is an iron–ligand type 5-LOX inhibitor that intercepts the active site iron and is the only redox inhibitor of 5-LOX approved for clinical use for the treatment of chronic bronchial asthma and allergic rhinitis and was prepared at a stock concentration of 10 mM in DMSO. ST1853 [32,33] was characterized as a new 5-LOX inhibitor and prepared at the same stock concentration as zileuton. These stock solutions were prepared in DMSO and stored at −20 °C. For experiments, different concentrations were used and diluted in DMEM. Zileuton was applied at final concentrations of 10 μM, and ST1853 was applied at concentrations of 0.5 μM. These concentrations were chosen on the basis of experiments where we determined concentration-dependent effects in neuronal HT22 cells.

For the 12/15-LOX inhibitor PD146176 (Sigma-Aldrich, Taufkirchen, Germany), a stock solution was prepared at a concentration of 5 mM, dissolved in DMSO, and stored at −20 °C. The concentration used was 5 µM.

Mitoquinone (MitoQ; Medkoo Biosciences, Morrisville, NC, USA) is a mitochondria-targeted form of Coenzyme Q10, a lipid-soluble antioxidant. Specific localization to mitochondria is facilitated by a lipophilic cationic carrier. Once accumulated in the mitochondrial matrix, MitoQ can scavenge ROS and free radicals in close proximity to their site of generation. The original stock of the mitochondria-targeted ubiquinone derivate was diluted in ethanol and water at a ratio of 1:1 (14.73 mM) and further diluted with DMSO to a stock concentration of 2 mM. MitoQ was used at final concentrations of 0.0625–0.5 µM.

The iron chelator deferoxamine (DFO; Sigma-Aldrich, Munich, Germany) was dissolved in DMSO to a stock concentration of 10 mM and stored at −20 °C. To inhibit cell death induced by RSL3 or to chelate iron, thereby limiting the generation of lipid peroxides and preventing ferroptotic cell death, the compound was further diluted in medium to a concentration of 2.5–10 μM.

Trolox (6-hydroxy-2,5,7,8tetramethyl-chroman-2-carboxylic acid) (Sigma-Aldrich, Taufkirchen, Germany) is a water-soluble analog of vitamin E and acts as a potent antioxidant. It scavenges ROS, including lipid peroxides, and reduces oxidative stress in the cell. The stock solution of Trolox (50 mM in DMSO) was further diluted to the use concentrations of 1–150 µM in DMEM medium.

Two different thiazolidinediones (TZDs) were used to specifically target and pharmacologically inhibit ACSL4. Troglitazone (TRO; TCI, Eschborn, Germany) was dissolved in DMSO to a stock concentration of 20 mM and further used in concentrations ranging from 0.1 to 1 µM, whereas the final concentration used was 0.25 µM. Rosiglitazone (ROSI; Cayman Chemical, Ann Arbor, MI, USA) was dissolved in DMSO to a stock concentration of 50 mM and further used in concentrations ranging from 1 to 75 µM, whereas the final concentration used was 25 µM.

To inhibit complex I of the respiratory chain, phenformin and metformin (Cayman Chemical, MI, USA) was used. Phenformin is a member of the biguanide compound family, which also includes metformin, which is widely used as an anti-hyperglycemic drug in the treatment of type-2 diabetes [34]. Originally diluted in H_2_O bidest, it was further diluted using DMSO to create stocks of 1, 10, and 100 mM and used in experiments at concentrations ranging from 5 to 20 μM. Metformin was dissolved in DMSO to a stock concentration of 1 M and was used in a concentration of 0.5–5 mM.

To inhibit the succinate dehydrogenase (SDH), referred to as complex II of the respiratory chain, itaconate (Sigma-Aldrich, Munich, Germany) was used. Itaconate occurs physiologically endogenously in macrophages, where it mediates anti-inflammatory regulation [35]. To generate an itaconate stock solution at a concentration of 250 mM, 1.6263 g itaconic acid was dissolved in DMEM culture medium, and the pH was adjusted to 7.34. In the experiments, itaconate was used at concentrations ranging between 5 and 20 mM. 4-octyl itaconate (4-OI; Sigma-Aldrich, Munich, Germany) is a less polar itaconate-derivative. 4-OI was dissolved in DMSO to a stock solution of 10 mM and was used at concentrations ranging from 0.5 to 2 µM.

GW9662 (Sigma-Aldrich, Munich, Germany) binds to the ligand binding site of peroxisome proliferator-activated receptor γ (PPARγ) and is therefore used as a PPARγ antagonist. GW1929 (Sigma-Aldrich, Munich, Germany) is described as a potent tyrosine-based PPARγ agonist. Both compounds were dissolved in DMSO to stock solutions of 1 and 10 mM and were used in concentrations ranging from 1 to 10 µM of GW9662 and 10 to 30 µM of GW1929.

### 2.2. RT-(q)PCR

Total RNA was isolated from HEK293T cells in 6-well plates 24 h after seeding, using the InviTrap Spin Universal RNA Mini Kit (Invitek Molecular GmbH, Berlin, Germany). For reverse transcription, PCR (RT-PCR) SuperScript III One-Step RT-PCR System (Thermo Fisher Scientific, Darmstadt, Germany) was used, as well as specific oligonucleotides as follows: human GAPDH (258 bp) forward 5′-AACAGCGACACCCACTCCTC-3′ and reverse 5′-GGAGGGGAGATTCAGTGTGGT-3′; ACSL4 (81 bp) forward 5′-TTTCAAACTGAAAAGGAAGGAGCT-3′ and reverse 5′-ACAACATTTTATTTGCCCCCAT-3′; and LPCAT2 (178 bp) forward 5′-TTGCTTCCAATTCGTGTCTTATT-3′ and reverse 5′-ATCCCATTGAAAAGAACATAGCA-3′. The amplification products were visualized by agarose gel electrophoresis after ethidium bromide staining and UV light illumination.

The StepOnePlus Real-Time PCR System (Fisher Scientific Gmbh, Schwerte, Germany) was used to conduct the quantitative PCR (qPCR). mRNA was extracted by using the InviTrap Spin Universal RNA Mini Kit according to the manufacturer’s protocol. mRNA was further treated with the Turbo DNA-Free Kit, and 200 ng of DNAse-treated RNA was used for cDNA synthesis, conducted with iScript^TM^ cDNA Synthesis Kit (Bio-Rad Laboratories GmbH, Munich, Germany). cDNA was then diluted with aqua bidest in a ratio of 1:5 and was then added to the master mix of iTaq Universal SYBR Green Supermix (Bio-Rad Laboratories GmbH, Munich, Germany), also containing the respective primer pair for the genes of interest and was afterward transferred into a MicroAmp^TM^ Fast Optical 96-Well Reaction Plate (Thermo Fisher Scientific, Waltham, MA, USA). Relative gene expression was calculated by the 2^−ΔΔCT^ method [36]. The oligonucleotides used for RT-qPCR were already described; in addition, the following U6 oligonucleotides were used: U6 forward (CTCGCTTCGGCAGCACA) and U6 reverse (AACGCTT CACGAATTTGCGT).

### 2.3. Protein Analysis and Western Blot

Protein extraction and visualization by Western blot analysis were performed as described before [21]. Briefly, after the respective treatments, cells in a 6-well plate were washed with PBS and then lysed using lysis buffer (0.25 M D-mannitol, 0.05 M Tris base, 1 mM EDTA, 1 mM EGTA, 1 mM DL-dithiothreitol (DTT), 1% Triton-X100 supplemented with protease, and phosphatase inhibitor cocktail tablets (Roche Diagnostics, Mannheim, Germany)). Afterward, the cell suspensions were centrifuged for 15 min at 10,000× *g* at 4 °C, and then the protein content was determined using Pierce BCA Protein Assay Kit (Perbio Science, Bonn, Germany). A total of 30–35 µg of proteins were separated by size by SDS-PAGE gel electrophoresis (12.5% SDS gels) and transferred on a polyvinylidenfluoride (PVDF) membrane (Roche Diagnostics, Mannheim, Germany) at 325 mA for 2.5 h. The primary antibodies used were as follows: vinculin (1:20,000 in 5% blocking buffer in TBST; Sigma-Aldrich, Taufkirchen, Germany) or actin (1:1500 in 5% blocking buffer; Novus Biologicals, Wiesbaden, Germany) as loading controls. ACSL4 (1:500 in 5% blocking buffer; Santa Cruz Biotechnology, Heidelberg, Germany), LPCAT2 (1:6000 in 5% blocking buffer; Proteintech, Planegg-Martinsried, Germany), GPx4 (1:500 in 5% blocking buffer, Abcam, Berlin, Germany), or xCT (1:50,000 in 5% blocking buffer, Proteintech, Planegg-Martinsried, Germany) were incubated overnight at 4 °C. Appropriate horseradish peroxidase-labeled secondary antibodies (1:2500 in 5% blocking buffer) were incubated for 1 h at room temperature on the next day before the detection of the Western blot signals by chemiluminescence with the Chemidoc system (Bio-Rad, Munich, Germany). Quantification was conducted using ImageLab software 6.1 (Bio-Rad, Munich, Germany).

### 2.4. Cell Viability Measurements

Cell viability was measured by a colorimetric assay based on the reduction in a yellow tetrazolium salt, MTT (3-(4,5-Dimethylthiazol-2-yl)-2,5-diphenyltetrazolium bromide; Sigma-Aldrich, Munich, Germany), to an insoluble purple-colored formazan product by dehydrogenases and NAD(P)H coenzyme of metabolically active cells as well as by mitochondrial enzymes [37]. The color of the purple formazan product correlates with metabolic activity. After treating HEK293T cells with RSL3 for an indicating time, 0.5 mg/mL MTT-solution was added to each well for 1 h incubation period at 37 °C. Afterward, the medium was completely removed to stop the reaction, and the 96-well plates were stored at −80 °C for at least 1 h. Then, the formazan was dissolved in 70 μL DMSO for 1 h on a shaker at 37 °C. Finally, absorbance was measured at 570 nm with a reference filter at 630 nm for background subtraction by FluoStar OPTIMA reader (BMG Labtech, Ortenberg, Germany).

The xCELLigence Real-Time Cell Analysis (RTCA; Roche Diagnostics, Mannheim, Germany) was used to reveal biological processes of living cells such as proliferation, cell growth, and cell death due to changes in cell morphology and attachment resulting in alterations of cell impedance as previously described [38]. Changes in the cell impedance are displayed as normalized cell index by RTCA Software 1.2.

### 2.5. Flow Cytometry

Different mitochondrial and cellular parameters were analyzed using the Guava easyCyte 6–2L system flow cytometer (Merck Millipore, Darmstadt, Germany). A total of 30 h before the treatment, 43,000–60,000 cells per well were seeded in a 24-well plate. After the indicated treatment time, adherent cells were harvested by washing them with PBS, followed by trypsinization. The detached cells were resuspended in medium, collected, and centrifuged at 2000 rpm for 5 min. The cell pellet was washed again with PBS, followed by the second centrifugation step. Thereafter, the cell pellet was stained with the respective fluorescent dye following the instructions for the different fluorescent dyes. The GuavaSoft Software 4.5 package was used for data analysis, and every experiment was performed with 5000 cells with at least three replicates per condition. For measurements of mitochondrial ROS accumulation, the cells were incubated with live-cell-permeable fluorescent sensor MitoSOX Red (Thermo Fisher Scientific, Darmstadt, Germany) at a concentration of 1.25 µM in PBS for 30 min at 37 °C in the dark and measured afterward (excitation: 488 nm; emission: 690/50 nm). For measuring the mitochondrial membrane potential, cells were stained with TMRE Kit (MitoPT, ImmunoChemistry Technologies, Hamburg, Germany) at a concentration of 0.2 µM TMRE (tetramethylrhodamine ethyl ester) for 30 min at 37 °C in the dark. TMRE fluorescence was measured utilizing excitation at 488 nm and emission at 690/50 nm. Cell death was detected using only propidium iodide (PI) (Promokine, Heidelberg, Germany) in HEK293T cells or PI and Annexin V FITC (Invitrogen, Karlsruhe, Germany) in HT22 cells, which stains early apoptotic cells. PI was used for the detection of late necrotic cells, which became permeable upon membrane disruption and allowed PI to pass through and bind to nucleic acids. PI staining was performed for 10–15 min in the dark at room temperature and measured afterward (excitation: 488 nm; emission: red filter, 690/50 nm; green filter, 525/30 nm).

### 2.6. ATP Bioluminescent Assay and Seahorse Measurement

The detection of cellular adenosinetriphosphate (ATP) is based on a bioluminescent assay, which utilizes the enzyme luciferase to catalyze the formation of light from ATP and luciferin. The emitted light is proportional to the ATP content in the respective sample and was detected by a plate reader (SPARK 20M, Tecan, Germany) measuring the luminescence. For the analysis of the cellular ATP level, the ViaLight Plus Kit (Lonza, Verviers, Belgium) was used according to the manufacturer’s protocol. Briefly, 7000–8000 cells were seeded in a 96-well plate 30 h before treatment, followed by the respective treatment. After the indicated treatment time, a lysis buffer was added to the cells and incubated for 10 min at room temperature. After the incubation time, 100 µL of each well was transferred into a white 96-well plate, and 100 µL AMR (ATP monitoring reagent) was added to each well, incubated for 2 min in the dark, and afterward, the luminescence was measured.

For determination of the mitochondrial oxygen consumption rate (OCR) as an indicator of respiration and extracellular acidification rate (ECAR) as an indicator of glycolysis, the Seahorse XFe96 Analyzer (Agilent Technologies, Waldbronn, Germany) was used. HEK293T cells were seeded in XFe96-well microplates (Agilent Technologies, Waldbronn, Germany), and one hour before measurement, the medium was replaced by the assay medium (2 mM glutamine, 1 mM pyruvate, and 4.5 g/L (25 mM) glucose, pH 7.35) and was then incubated for 60 min at 37 °C without CO_2_. Three baseline measurements were recorded, followed by injections of four compounds. The used compounds and final concentrations were as follows: injection A, 3 µM oligomycin (oligo) (ATP synthase inhibitor; Merck KGaA, Darmstadt, Germany); injection B, 0.5 µM Carbonyl cyanide-4-(trifluoromethoxy)phenylhydrazone (FCCP) (uncoupling agent; Merck KGaA, Darmstadt, Germany); injection C, 0.1 µM rotenone and 1 µM antimycin A (AA) (complex I/III inhibitors; Merck KGaA, Darmstadt, Germany); and injection D, 50 mM 2-deoxyglucose (2-DG) (glycolysis inhibitor; Carl Roth GmbH, Karlsruhe, Germany). After the injection of each compound, three measurements were performed with 4 min mixing, followed by 3 min detection. For evaluation, the data were normalized to the respective cell density, determined as the protein content of each well by Pierce BCA kit (Perbio Science, Bonn, Germany).

### 2.7. Glutathione Assay

For the determination of the glutathione levels, 300,000–400,000 HEK293T cells were seeded in 6-well plates 24–30 h before treatment. The treatment was carried out in triplicate, and after the indicated treatment time, the cells were harvested in growth medium, where triplicates were pooled. After centrifugation, the cells were washed with PBS, followed by the second centrifugation step. The supernatant was removed, and cell pellets were frozen in N_2_ and stored at −80 °C until use. The Glutathione Assay Kit (Cayman Chemical Company, Ann Arbor, MI, USA) was used according to the manufacturer’s instructions. Briefly, cell pellets were resuspended and sonicated in MES buffer (0.4 M 2-(N-morpholino) ethanesulfonic acid, 0.1 M phosphate, and 2 mM EDTA, pH 6.0). After centrifugation at 10,000× *g* for 15 min at 4 °C, the supernatant was removed, and afterward, 1.25 M metaphosphoric acid (MPA) (Sigma-Aldrich, Taufkirchen, Germany) was added to the supernatant, incubated for 5 min at room temperature for deproteination and then centrifuged at 17,000× *g* for 10 min. The supernatant was carefully collected without disturbing the precipitate, and the pH was increased using a 4 M triethanolamine solution (Sigma-Aldrich, Taufkirchen, Germany). The samples were transferred into a 96-well plate in duplicate, where the freshly prepared assay cocktail was added and incubated for 25 min in the dark at room temperature. Afterward, the absorbance was measured at 405 nm by a plate reader (SPARK 20M, TECAN, Germany) and corrected thereafter. The total GSH amount was calculated via the standard curve and normalized to protein content after the BCA assay.

### 2.8. DPPH Assay

For the determination of the radical scavenging activity of the used compounds, the 2,2-diphenyl-1-picrylhydrazyl (DPPH) assay (Cayman Chemical Company, Ann Arbor, MI, USA) was used. The compounds and Trolox, which serves as a reference substance due to its pronounced antioxidant activity, were prepared in 75% ethanol to the respective concentrations of. In a 96-well plate, 90 µL of DPPH solution (150 µM) with 10 µL of the compound were mixed and incubated in the dark for 30 min. At least 3 wells per condition were used. A total of 50 µM, 100 µM, or 150 µM of Trolox was used as positive control, and 75% ethanol and DMSO as negative control. Absorbance was measured at 517 nm by a plate reader (SPARK 20M, Tecan, Germany), and the DPPH scavenging effect was calculated with the following formula: $\frac{A_{0}-A_{x}}{A_{0}}\times 100$. $A_{0}$ represents the absorbance of ethanol, and $A_{x}$ the absorbance of the individual substances.

### 2.9. Statistical Analysis

For statistical calculations, the software GraphPad Prism 9 (San Diego, CA, USA) and Winstat standard statistical software version 2007 (R. Fitch Software, Witzenhausen, Germany) were used. For multiple comparisons, ANOVA was performed in combination with Scheffé’s post hoc-test or Bonferroni. Data with *p* < 0.001 (***/###/+++), *p* < 0.01 (**/##), and *p* < 0.05 (*/#) are considered significantly different. No indication of significance signifies not significant.

## 3. Results

### 3.1. ACSL4 and LPCAT2 Overexpression Increases the Responsiveness of HEK293T Cells to Ferroptosis

**Figure 1:**
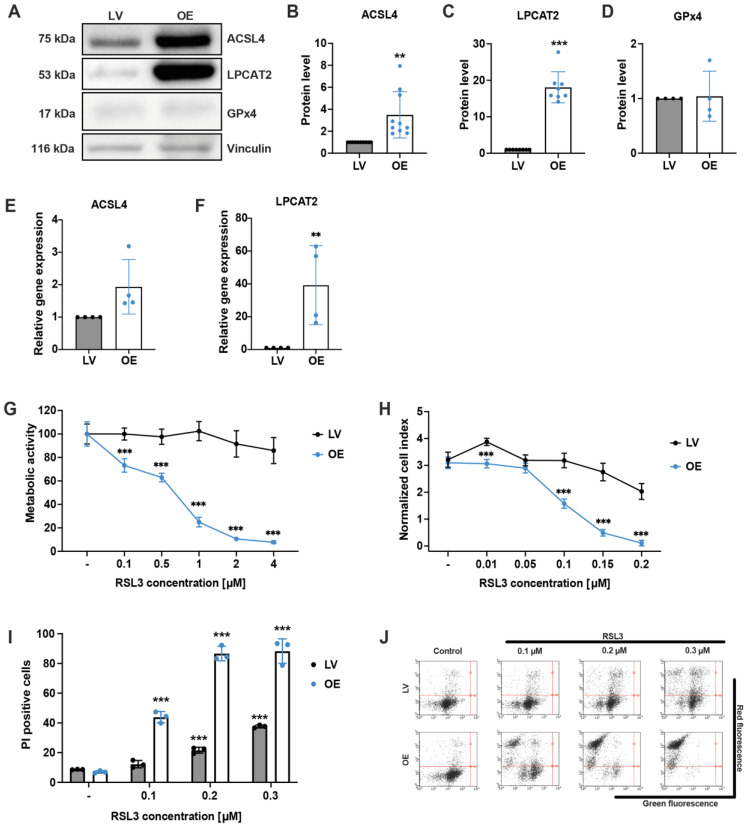
Quantification of protein and mRNA levels and sensitivity of empty vector control and ACSL4/LPCAT2 overexpressing HEK293T cells against RSL3. (**A**–**D**) Overexpression of ACSL4 and LPCAT2 by the sleeping beauty system was confirmed by (**A**) Western blot analysis, which was quantified for protein levels (**B**) ACSL4 (*n* = 10) (**C**) LPCAT2 (*n* = 8) and (**D**) GPx4 (*n* = 4) and depicted as fold protein level normalized to controls. (**E**,**F**) Quantitative PCR analysis of untreated LV and ACSL4/LPCAT2 OE cells. Relative gene expression was calculated for (**E**) ACSL4 and (**F**) LPCAT2 by normalization to GAPDH RNA level and LV control conditions (data are given as individual data points ± SD; *n* = 4 replicates per group). Sensitivity against RSL3 was analyzed by (**G**) MTT assay after 16 h of RSL3 treatment (percentage of control condition) and (**H**) xCELLigence real-time impedance measurement evaluated 19 h after treatment onset. Data are given as mean ± SD (*n* = 8 replicates). (**I**) Cell death was quantified by FACS analysis of PI staining after 16 h of RSL3 treatment (5000 cells per replicate of *n* = 3 replicates, percentage of gated cells). (**J**) Representative dot plots of the FACS measurements. *** *p* < 0.001; ** *p* < 0.01 compared to (untreated) control (ANOVA, Scheffé’s test).

HEK293T cells were stably transfected using the sleeping beauty system [30] with an empty vector or overexpression of the enzymes ACSL4 and LPCAT2, resulting in an average of 3.5-fold overexpression of ACSL4 and 18-fold overexpression of LPCAT2 detected in Western blot analyses (Figure 1A–C). The protein expression levels of the selenoprotein GPx4 (Figure 1D) and the system Xc^−^ (Supplementary Figure S1A,B) were not altered in OE cells compared to LV controls. Relative gene expression levels of ACSL4 and LPCAT2 were quantified by qPCR, revealing 2-fold higher mRNA levels of ACSL4 and, on average, 40-fold higher LPCAT2 levels in the overexpressing cells compared with empty vector control cells (Figure 1E,F). To evaluate the effects of the ACSL4/LPCAT2 overexpression on cell viability, both cell lines were treated with different concentrations of the GPx4 inhibitor RSL3 reaching from 10 nM to 4 µM. The MTT assay illustrates a significant reduction in the metabolic activity after 16 h of RSL3 exposure in the OE cells, even at the lowest applied RSL3 concentration of 100 nM, whereas LV cells remained largely unchanged (Figure 1G). These results were confirmed by real-time impedance measurements (Figure 1H and Supplementary Figure S2A) and by detecting cell death through propidium iodide (PI) staining and flow cytometry (Figure 1I,J), demonstrating that the OE cell line was considerably more sensitive to RSL3 compared to the control cell line, due to the overexpression of the ACSL4 and LPCAT2. The LV cells also responded to the ferroptosis inducer, however, at considerably higher concentrations of RSL3 and with reduced sensitivity compared to the ACSL4/LPCAT2 OE cell line. It should be noted that the required concentrations of RSL3 to induce ferroptosis varied due to density or passage-dependent differences that affected the responsiveness of the cells to the ferroptosis stimulus between the experiments. However, the relative difference in sensitivity to ferroptosis between OE and LV cells was very robust and highly reproducible and served as a consistent and reliable reference point for comparison, irrespective of the specific RSL3 concentrations used.

### 3.2. Ferroptosis Inhibitors Deferoxamine and Ferrostatin-1 Protect against RSL3-Mediated Cell Death

**Figure 2:**
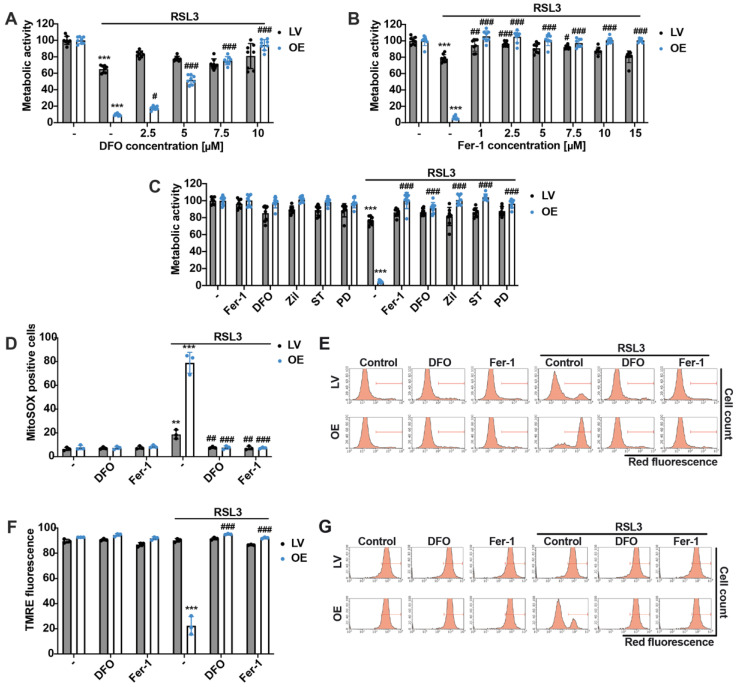
DFO and Fer-1 prevent ACSL4/LPCAT2 OE cells from increased mitochondrial ROS production and loss of mitochondrial membrane potential and metabolic activity. Metabolic activity of HEK293T cells was evaluated by MTT assays after 16 h of treatment with (**A**) 0.8 µM RSL3 and co-treatment with 2.5–10 µM deferoxamine (DFO); (**B**) 0.18 µM RSL3 and co-treatment with 1–15 µM ferrostatin-1 (Fer-1); and (**C**) 0.2 µM RSL3 and co-treatment with 10 µM Fer-1, 10 µM DFO, 10 µM zileuton (Zil), 0.5 µM ST1853 (ST) and 5 µM PD146176 (PD). Data are shown as percentage of control condition of *n* = 8 replicates. (**D**) Mitochondrial ROS formation and (**F**) mitochondrial membrane potential were quantified by FACS analysis of MitoSOX or TMRE stained cells after 0.2 µM RSL3 and co-treatment with 10 µM DFO and Fer-1 for 16 h (5000 cells per replicate of *n* = 3 replicates, percentage of gated cells). (**E**,**G**) Histograms of the respective FACS measurements with gating. *** *p* < 0.001; ** *p* < 0.01 compared to control condition, ### *p* < 0.001; ## *p* < 0.01; and # *p* < 0.05 compared to RSL3-treated control condition (ANOVA, Scheffé’s test).

In order to prove that the observed RSL3-induced cell damage was indeed mediated through ferroptosis in the HEK293T cell lines, both deferoxamine (DFO) and the ferroptosis inhibitor ferrostatin-1 (Fer-1) were tested in the present model system of RSL3 induced cell death. DFO acts as an iron chelator, and Fer-1 has been described as a specific and potent inhibitor of ferroptosis by inhibiting RSL3-induced lipid peroxidation [1]. DFO showed concentration-dependent protective effects, with maximum efficacy at 10 µM when co-treated with RSL3 for 16 h in the MTT assay (Figure 2A). Also, Fer-1 completely prevented ferroptosis in both HEK293T cell lines (Figure 2B), even at low concentrations of 0.1 µM (Supplementary Figure S3A). Moreover, three different LOX inhibitors were tested to investigate LOX-dependent ferroptosis in this model system. The 5-LOX inhibitors zileuton and ST1853 and the 12/15-LOX inhibitor PD146176 were able to protect ACSL4/LPCAT2 overexpressing HEK293T cells from oxidative cell death to the same extent as DFO and Fer-1 (Figure 2C), and Trolox, a lipophilic antioxidant, also exerted protective effects (Supplementary Figure S3B), confirming that RSL3 induced cell death was mediated via ferroptosis. In addition, protective effects could be detected on mitochondrial parameters like mitochondrial ROS formation (Figure 2D,E) and mitochondrial membrane potential (Figure 2F,G) when co-treating 0.2 µM RSL3 with 10 µM of the ferroptosis inhibitors for 16 h. These results indicated that the ferroptosis inhibitors DFO and Fer-1 abrogated mitochondrial impairment after ferroptosis-induction by RSL3.

### 3.3. Pharmacological ACSL4 Inhibition by Thiazolidinediones Prevented Ferroptosis in ACSL4/LPCAT2 Overexpressing Cells

**Figure 3:**
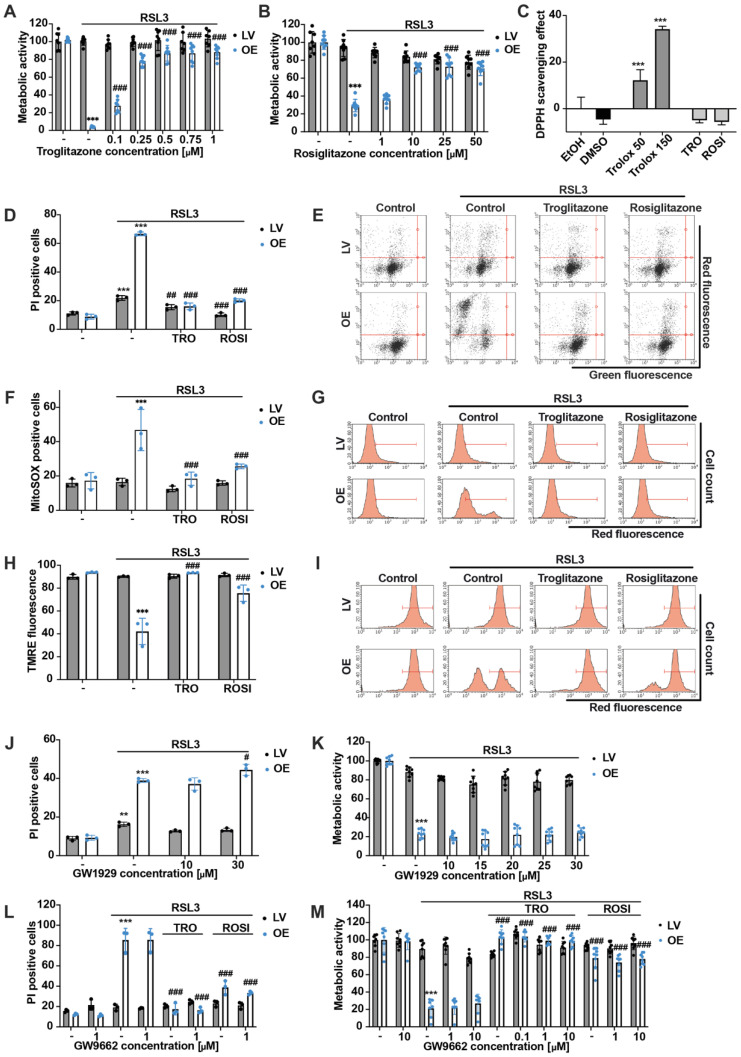
Troglitazone and Rosiglitazone inhibit ferroptosis in ACSL4/LPCAT2 overexpressing cells. (**A**,**B**) Metabolic activity was determined by MTT assay after 16 h of exposure to 0.1 µM or 0.5 µM RSL3 and co-treatment with 0.1–1 µM troglitazone (TRO) and 1–50 µM rosiglitazone (ROSI). Data are shown as percentage of control conditions of *n* = 8 replicates. (**C**) For the determination of antioxidant properties of the substances, a DPPH assay was performed with 50 µM and 150 µM Trolox, 0.25 µM TRO, and 25 µM ROSI. Data are given as mean ± SD (*n* = 3 replicates). (**D**) Cell death, (**F**) mitochondrial ROS formation, and (**H**) mitochondrial membrane potential were quantified by FACS analysis of PI, MitoSOX, or TMRE stained cells after co-treatment of 0.25 µM TRO and 25 µM ROSI with 0.1, 0.15, or 0.5 µM RSL3 for 16 h (5000 cells per replicate of *n* = 3 replicates, calculated as percentage of gated cells). (**E**,**G**,**I**) Representative dot plots or histograms of the respective FACS measurements with gating. Cell death measured by PI staining after 16 h treatment of (**L**) 1 µM RSL3 co-treated with 1 µM GW9662, 0.25 µM TRO, and 25 µM ROSI and (**J**) 0.1 µM RSL3 co-treated with 10 µM and 30 µM GW1929 (5000 cells per replicate of *n* = 3 replicates, percentage of gated cells). MTT assays of HEK293T cells after 16 h treatment with (**M**) 0.6 µM RSL3 and co-treated with 0.1, 1, or 10 µM GW9662 and 0.25 µM TRO or 25 µM ROSI and (**K**) 1 µM RSL3 with 10–30 µM GW1929 were evaluated. Data are shown as percentage of control conditions, *n* = 8 replicates. *** *p* < 0.001; ** *p* < 0.01 compared to control conditions, ### *p* < 0.001; ## *p* < 0.01; and # *p* < 0.05 compared to RSL3-treated control cells (ANOVA, Scheffé’s test).

We next applied pharmacological inhibition of ACSL4 to confirm that the ACSL4 overexpression and the subsequently active enzyme LPCAT2 mediated the enhanced sensitivity of OE cells to ferroptosis. Recently, thiazolidinediones (TZDs) have been reported to specifically block ACSL4 compared to other ACSL isoforms [39], and therefore, the two TZD compounds troglitazone (TRO) and rosiglitazone (ROSI) were tested in the LV and OE HEK293T cells. First, the inhibitors were titrated for their protective concentrations in the MTT assay when co-treated with the ferroptosis inducer RSL3 (Figure 3A,B). In addition, the antioxidant properties of the TZDs were checked by performing a DPPH assay at the protective concentrations of 0.25 µM troglitazone and 25 µM rosiglitazone (Figure 3C). The DPPH assay confirmed that the observed protection was not attributable to the antioxidant properties of the substances. In addition, the protective effect against RSL3-induced cell death by pharmacological ACSL4 inhibition was also confirmed by PI staining (Figure 3D,E). Further, parameters of mitochondrial impairment, such as mitochondrial ROS accumulation (Figure 3F,G) and the loss of mitochondrial membrane potential (Figure 3H,I) were prevented by both TZDs. Since the TZD compounds TRO and ROSI were also described as a class of peroxisome proliferator-activated receptor-γ (PPARγ) agonists [40], the non-TZD PPARγ agonist GW1929 was applied in LV and OE cells, and neither showed protective effects in the MTT assay (Figure 3K) nor in flow cytometry after PI staining (Figure 3J). To further confirm the protective effects of TRO and ROSI against ferroptosis independent of PPARγ modulation, both inhibitors were combined with the PPARγ-antagonist GW9662 [41]. As shown in Figure 3, GW9662 neither affected the protective effects of troglitazone nor those of rosiglitazone after RSL3 treatment as measured by FACS analysis after PI staining (Figure 3L) and in the MTT assay (Figure 3M), confirming that the effects of the TZD ACSL4 inhibitors were achieved independent of PPARγ. Consequently, the increased sensitivity of OE cells to the ferroptosis insult resulted from the overexpression of ACSL4 and LPCAT2, and specific pharmacological inhibition of ACSL4 preserved the cells from RSL3-mediated ferroptosis and prevented the accompanying detrimental effects on the mitochondria.

### 3.4. ACSL4 and LPCAT2 Overexpression Causes Massive Mitochondrial Impairment

**Figure 4:**
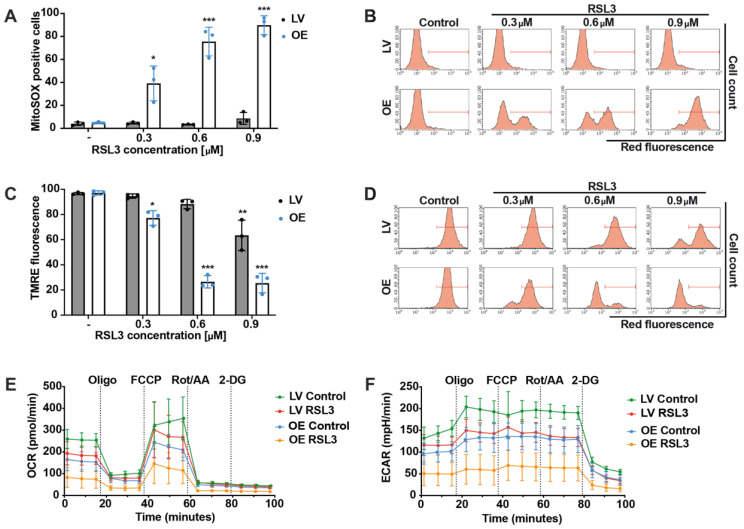
FACS analysis and Seahorse measurement demonstrate mitochondrial involvement in ACSL4 and LPCAT2 OE cells. (**A**) Mitochondrial ROS formation and (**C**) mitochondrial membrane potential were quantified by FACS analysis of MitoSOX or TMRE stained cells after 0.3, 0.6, and 0.9 µM RSL3 treatment for 16 h (5000 cells per replicate of *n* = 3 replicates, calculated as percentage of gated cells). (**B**,**D**) Representative histograms of the respective FACS measurement with gating. (**E**) Mitochondrial respiration and (**F**) glycolysis were detected by the seahorse system after 16 h of 0.8 µM RSL3 treatment (*n* = 6–8 replicates per condition). *** *p* < 0.001; ** *p* < 0.01; and * *p* < 0.05 compared to untreated control conditions (ANOVA, Scheffé’s test).

Based on the previous findings, we were interested in assessing if, under conditions of ACSL4/LPCAT2 overexpression, mitochondria were significantly involved in the enhanced sensitivity of the HEK293T cells against ferroptosis. For this purpose, we used the MitoSOX marker for specific detection of mitochondrial superoxide formation. In line with the effects on cell viability, RSL3 significantly enhanced mitochondrial ROS production in OE cells. In contrast, mitochondrial ROS accumulation was not detected in the LV cells after 16 h of RSL3 treatment (Figure 4A,B). When measuring mitochondrial membrane potential using the fluorescent dye TMRE, a slight decrease in MMP was detected at the highest RSL3 concentration in the LV control cells. Nevertheless, the OE cells revealed a massive loss of mitochondrial membrane potential after exposure to RSL3 (Figure 4C,D). For the detection of energy metabolism, the Seahorse XFe96 analyzer was used to quantify mitochondrial respiration by measuring the oxygen consumption rate (OCR) as well as glycolysis by detecting the extracellular acidification rate (ECAR). These measurements displayed that the ACSL4 and LPCAT2 overexpressing cells exerted slightly lower basal respiration and glycolysis rates in comparison to control cells (Figure 4E,F). RSL3 treatment resulted in a reduced mitochondrial respiration and glycolysis rate in both cell lines, with the OE cells exhibiting a more pronounced decrease in both ECAR and OCR after 16 h of treatment compared to LV controls. These results imply that mitochondria play an essential role in RSL3-mediated ferroptosis in ACSL4/LPCAT2 overexpressing HEK293T cells, and that increased sensitivity to ferroptosis was also associated with severe mitochondrial damage.

### 3.5. Mitochondrial ROS Scavenger Mitoquinone Prevents RSL3-Mediated Ferroptosis in ACSL4/LPCAT2 OE Cells

**Figure 5:**
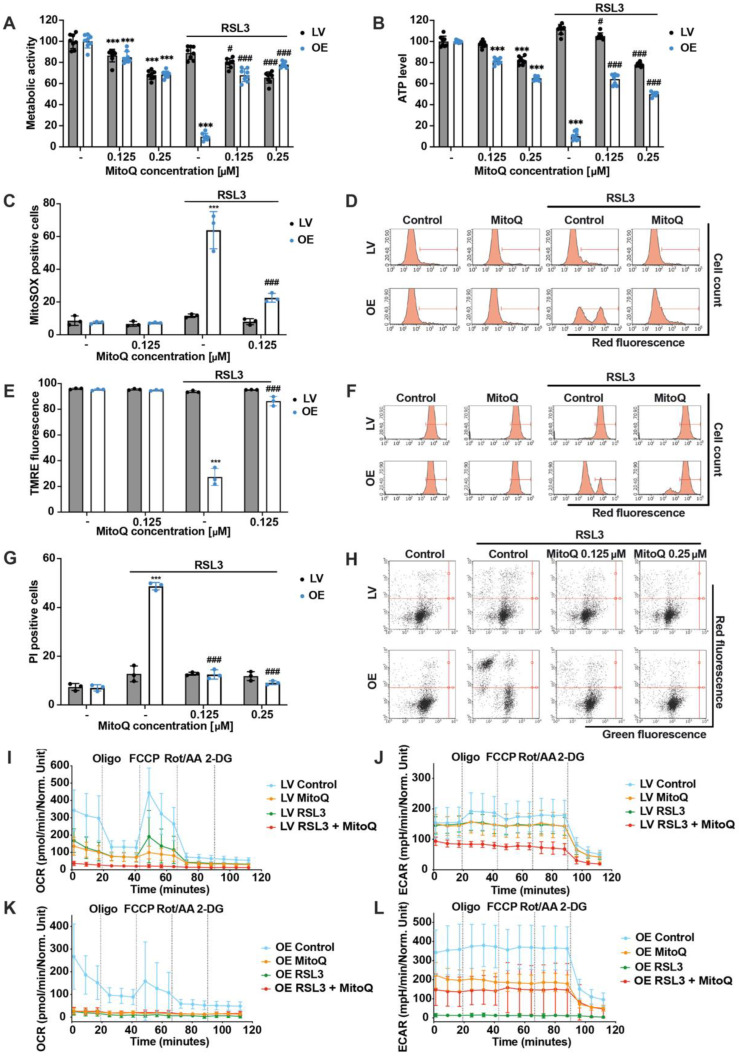
Mitochondrial ROS scavenging by mitoquinone prevents RSL3-mediated ferroptosis in ACSL4/LPCAT2 OE cells. (**A**) Metabolic activity and (**B**) ATP levels were determined by MTT or ATP assay after 16 h treatment with 0.1 µM RSL3. Data are shown as percentage of control conditions, *n* = 8 replicates. (**C**) Mitochondrial ROS formation, (**E**) mitochondrial membrane potential, and (**G**) cell death were quantified by FACS analysis of MitoSOX, TMRE, or PI-stained cells after co-treatment with 0.125 µM or 0.25 µM mitoquinone (MitoQ) and 0.4 µM (MitoSOX) or 0.8 µM RSL3 (TMRE, PI) for 16 h (5000 cells per replicate of *n* = 3 replicates, percentage of gated cells). (**D**,**F**,**H**) Representative histograms, or dot plots of the respective FACS measurements with gating. (**I**,**K**) Mitochondrial respiration and (**J**,**L**) glycolysis measurements by the seahorse XF analyzer of LV (**I**,**J**) and OE (**K**,**L**) cells treated with 0.4 µM RSL3 and co-treated with 0.25 µM MitoQ for 16 h (*n* = 5–8 replicates per condition). *** *p* < 0.001 compared to control condition, ### *p* < 0.001; # *p* < 0.05 compared to RSL3-treated control condition (ANOVA, Scheffé’s test).

Intrigued by our findings that mitochondrial impairment was detected in ACSL4/LPCAT2-driven ferroptosis, we further investigated the role of mitochondria in ferroptosis execution in these cells. As mentioned before, mitochondria have been identified as a major source of ROS, thereby acting as an amplification system and key decision point in ferroptosis. Thus, the mitochondria-targeted ROS scavenger MitoQ was added to block mitochondrial ROS accumulation and propagation of oxidative cell death [8,42,43]. As shown in the MTT assay, the cells maintained metabolic activity after RSL3 addition by co-treating low concentrations as 0.125 µM MitoQ (Figure 5A). In addition, ATP levels of ACSL4/LPCAT2-overexpressing HEK293T cells were preserved as well by the co-treatment of the mitochondrial ROS scavenger despite RSL3 exposure (Figure 5B). MitoQ concentrations exerted a minor concentration-dependent inhibitory effect on metabolic activity and ATP production in HEK293T cells at basal levels, whereas this does not reflect cytotoxic activity, as seen by the cell death measurement (Supplementary Figure S4A). Further, full protection by MitoQ was detected for mitochondrial ROS production (Figure 5C,D), mitochondrial membrane potential (Figure 5E,F), and cell death (Figure 5G,H). Also, lower MitoQ concentrations were tested by measuring PI-positive cells after challenging the HEK293T cells for 6 h with RSL3, revealing 0.125 µM MitoQ as the lowest concentration to achieve full protection, while EC50 values were detected at MitoQ concentrations of 0.0625 µM (Supplementary Figure S4B). Lastly, the effects of 0.25 µM MitoQ on mitochondrial respiration and glycolysis were measured using the Seahorse XFe96 analyzer, demonstrating reduced OCR after exposure to MitoQ in both cell lines, which was entirely depleted by co-treatment with RSL3, indicating that basal mitochondrial respiration, as well as the reserve capacity, were inhibited by MitoQ, and no response to the potent uncoupler FCCP could be detected (Figure 5I,K). Similar responses were detected for glycolysis of LV cells: MitoQ and RSL3 slightly reduced the ECAR compared to the untreated controls, whereas the simultaneous application of MitoQ and RSL3 significantly decreased the ECAR (Figure 5J). The glycolytic rate in OE cells was increased compared to the LV cells under basal conditions, but this was also reduced by MitoQ. In the OE cells, the induction of ferroptosis by RSL3 resulted in a severe shutdown of glycolysis. However, MitoQ co-treatment maintained the energy supply through glycolysis, with simultaneous inhibition of mitochondrial respiration and concomitant reduction in mitochondrial ROS production (Figure 5K,L). In striking contrast, RSL3-treated OE cells failed to maintain their glycolytic activity, reflecting a breakdown of energy metabolism in the cells undergoing ferroptotic death. In summary, these results indicate that the mitochondria-targeted antioxidant property of MitoQ rescues OE cells from ferroptosis and highlights a crucial role of mitochondria in ACSL4/LPCAT2-driven ferroptosis.

### 3.6. Inability to Protect ACSL4/LPCAT2-Driven Ferroptosis via Metabolic Intervention by Metformin

**Figure 6:**
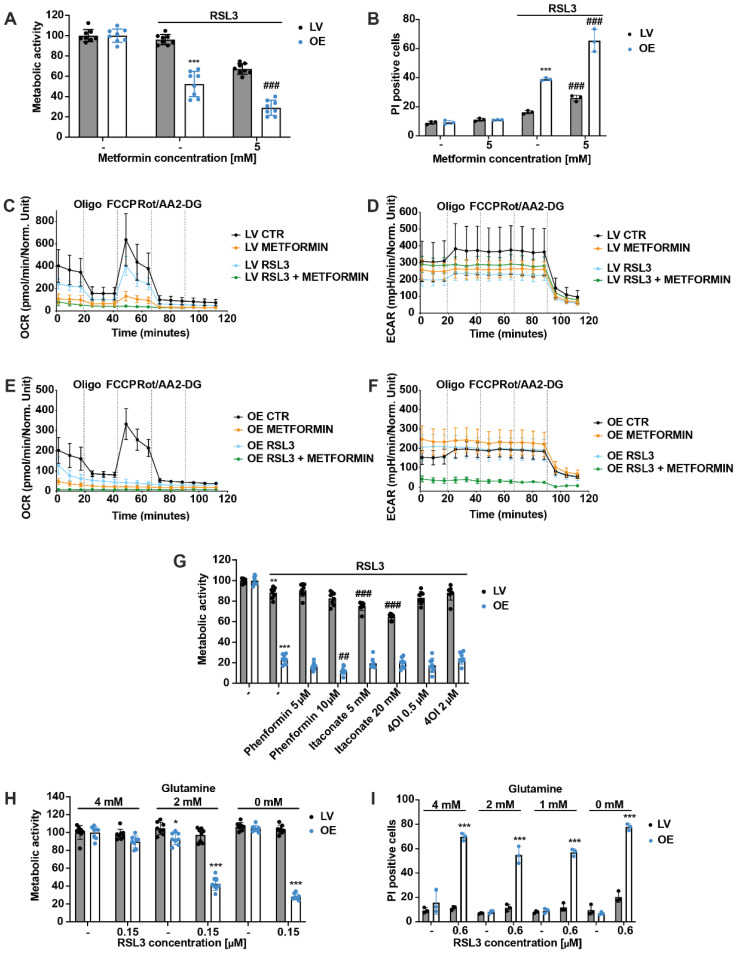
Metabolic intervention fails to prevent ferroptosis in ACSL4/LPCAT2 OE cells. (**A**) Metabolic activity was determined by MTT assay after 16 h treatment with 1 µM RSL3 and co-treatment with 5 mM metformin. Data are shown as percentage of control conditions, *n* = 8 replicates. (**B**) Cell death was measured by PI staining after treating HEK293T cells with 0.1 µM RSL3 and 5 mM metformin for 16 h (5000 cells per replicate of *n* = 3 replicates, percentage of gated cells). (**C**,**E**) Mitochondrial respiration and (**D**,**F**) glycolysis measurements by the Seahorse XF analyzer of LV (**B**,**C**) and OE (**D**,**E**) cells treated with 1 µM RSL3 and co-treated with 5 mM metformin for 16 h (*n* = 5–8 replicates per condition). (**G**) MTT assay after 16 h treatment with 1 µM RSL3 and co-treatment with 0.5 or 2 µM 4-octyl-itaconate (4OI), 5 or 10 µM phenformin, and 5 or 20 mM itaconate (Data are shown as percentage of control condition, *n* = 8 replicates). (**H**) Metabolic activity was determined by MTT assay after 16 h of exposure to 0.15 µM RSL3 in DMEM medium containing 4, 2, or 0 mM glutamine. Data are shown as percentage of control conditions of *n* = 8 replicates. (**I**) To confirm the MTT results, cells were measured by PI staining 18 h after treatment with 0.6 µM RSL3 under conditions of 4, 2, 1, or 0 mM glutamine in DMEM medium (5000 cells per replicate of *n* = 3 replicates, percentage of gated cells). *** *p* < 0.001; ** *p* < 0.01; and * *p* < 0.05 compared to control conditions and ### *p* < 0.001; and ## *p* < 0.01 compared to RSL3-treated control cells (ANOVA, Scheffé’s test).

Since scavenging of mitochondrial ROS production and protection against ACSL4/LPCAT2-driven ferroptosis by MitoQ was associated with reduced mitochondrial respiration and shift of energy metabolism towards glycolysis, we aimed to determine whether metabolic intervention alone, i.e., independent of any antioxidant properties, would be sufficient for protection against ferroptosis in the OE cells. We, therefore, applied metformin to shift energy metabolism from mitochondrial OXPHOS to an increase in aerobic glycolysis. Surprisingly, metformin reduced metabolic activity in RSL3-treated cells to a greater extent compared to RSL3 treatment alone in both cell lines (Figure 6A), which was confirmed by the cell death measurement even showing enhanced cell death after metformin treatment instead of protection (Figure 6B). Furthermore, metformin decreased OCR in the LV control cells under basal culture conditions, and OCR was completely shut down after co-treatment of metformin and RSL3 (Figure 6C). Again, the OE cells were even more responsive to metformin than LV cells, resulting in a total deactivation of the respiration by metformin already under basal culture conditions (Figure 6E). The ECAR of the LV cells slightly increased after co-administration of metformin and RSL3, compared to the RSL3 treatment alone (Figure 6D). Treating the OE cells with the ferroptosis inducer did not change the glycolytic rate, whereas co-treatment with metformin downregulated glycolysis (Figure 6F). Overall, metabolic intervention by metformin not only failed to prevent ACSL4/LPCAT2-driven ferroptosis but exacerbated mitochondrial respiratory impairment and cell death. In addition, phenformin, another biguanide agent, was tested in this model system, also failing to exert any protective effects (Figure 6G and Supplementary Figure S5). Another attempt was to inhibit the succinate dehydrogenase (SDH), i.e., complex II of the respiratory chain, with the compounds itaconate and the cell-permeable itaconate derivative 4-octyl itaconate (4OI). However, no effects were detected with the complex II inhibitors either (Figure 6G). Finally, because glutamine is known to be pivotal for mediating mitochondrial pathways of ferroptosis through metabolic effects [44], LV and OE cell lines were challenged with the ferroptosis inducer RSL3 in conditions of glutamine deprivation. The results showed that under basal conditions of glutamine deprivation, the metabolic activity of the cells was unaffected, whereas after RSL3 treatment, reduced glutamine concentrations resulted in significantly decreased metabolic activity in the OE cells (Figure 6H). Moreover, glutamine deprivation did not attenuate ferroptosis in ACSL4/LPCAT2-OE HEK293T cells (Figure 6I), indicating that glutaminolysis and the accompanying metabolic activity in mitochondria were not required in ACSL4/LPCAT2-driven ferroptosis in the HEK293T cells.

## 4. Discussion

ACSL4 and LPCAT accelerate ferroptosis by providing the required fatty acid and oxylipin substrates, leading to excessive lipid peroxidation [10,19]. Using HEK293T cells overexpressing ACSL4 and LPCAT2, we confirmed the important role of these enzymes for the sensitivity of the HEK293T cells to RSL3-induced ferroptosis and found an extensive mitochondrial involvement in ferroptotic cell death demonstrated through impaired mitochondrial respiration and reduced ATP production, disturbed mitochondrial membrane potential, and increased mitochondrial ROS formation. Mitochondrial demise and oxidative death through ferroptosis could be prevented by ferroptosis inhibitors such as Fer-1, 5- and 12/15-LOX inhibitors, ACSL4 inhibitors, the iron-chelator DFO and the lipophilic antioxidant Trolox, as well as by mitochondria-targeted ROS-scavenging in the OE cells. In contrast, metabolic intervention through inhibition of mitochondrial respiration by metformin or glutamine deprivation was insufficient to block ferroptosis. Thus, ACSL4 and LPCAT2 overexpression resulted in an increased susceptibility towards RSL3-induced oxidative cell death that required mitochondrial ROS formation downstream of the accelerated lipid peroxidation.

It is worth considering the potential impact of overexpressing ACSL4 and LPCAT2 on the physico-chemical features of the cell membrane. ACSL4 and LPCAT2 enzymes accelerate ferroptosis by promoting the incorporation of PUFAs into cell membranes, leading to increased susceptibility to lipid peroxidation [10]. The accumulation of lipid peroxides and the generation of oxylipins contribute to membrane damage and oxidative stress, ultimately leading to ferroptotic cell death. The upregulation of these enzymes may lead to an increased abundance of lipids containing unsaturated fatty acids within the membrane, which can affect its fluidity. Unsaturated fatty acids introduce structural changes in the lipid bilayer, rendering it more flexible and fluid compared to membranes enriched in saturated fatty acids. Consequently, the enhanced membrane fluidity resulting from ACSL4 and LPCAT2 overexpression may have implications for membrane dynamics, protein–lipid interactions, and overall membrane stability and may also influence the susceptibility of the cells to various cellular processes. However, it is still unclear to what extent it influences ferroptosis. The dysregulation of lipid metabolism involving ACSL4 and LPCAT2 has emerged as a critical regulatory mechanism in ferroptosis, and understanding these processes opens potential avenues for the development of therapeutic strategies targeting ferroptotic cell death in various diseases.

The widely used ferroptosis inhibitors Fer-1 and DFO, as well as 5- or 12/15-LOX-inhibitors, prevented RSL3-mediated cell death in the OE cells, confirming that ACSL4/LPCAT2 overexpression accelerated ferroptosis in this model system. This is in line with findings by Dixon et al., who identified that the genes ACSL4 and LPCAT3 are crucial for ferroptosis [16], and findings reported by Doll et al., who described that specifically the isoform ACSL4, over other family members of ACSL, influences the required lipid composition for ferroptosis [10]. Despite low 5-LOX expression and non-detectable 5-LOX activity in the HEK293T cells, the applied 5-LOX inhibitors ST1853 and zileuton exhibited protective effects against RSL3-mediated ferroptosis in the present study. This may be due to non-specific LOX inhibition or because the compounds exerted protective effects through alternative redox protection mechanisms, such as lipid peroxide scavenging. In the DPPH assay, however, no radical scavenging potential of the applied 5-LOX inhibitors was detected, whereas the lipid ROS scavenger and ferroptosis inhibitor Trolox showed full radical scavenging activity in this assay (Supplementary Figure S6). Nevertheless, it cannot be excluded that both 5-LOX inhibitors may exert redox-regulation and protective activities through other mechanisms affecting the iron pool or lipid ROS scavenging in the ferroptotic cells. Further, pharmacological inhibition of ACSL4 by the TZDs (troglitazone and rosiglitazone) reversed the elevated sensitivity of the overexpressing cells against RSL3-mediated ferroptosis, confirming that ACSL4 overexpression was mainly responsible for the enhanced susceptibility towards RSL3-mediated ferroptosis. Therefore, it can be concluded that the ACSL4 enzyme is essential for the oxidative cell death pathway, which is consistent with the findings of Yuan et al., showing that ACSL4 is responsible for lipotoxicity through the synthesis of 5-LOX-derived oxylipins and initiation of ferroptosis [14]. Since the applied TZDs are classified as PPARγ agonists in addition to their ACSL4 inhibitory effects [40], we applied compounds addressing PPARγ independent of ACSL4 inhibition. Treatment with the PPARγ agonist GW1929 or the PPARγ antagonist GW9662 showed that the agonist did not rescue the OE cells from ferroptosis, and the antagonist does not diminish the effects of the TZDs. These observations are in line with findings by Conrad and colleagues, also demonstrating the selectivity of the TZDs on ACSL4, which further supports the conclusion that the detected effects were mediated through inhibition of ACSL4 and independent of PPARγ [10]. Additionally, the authors suggested that the antioxidant effects of troglitazone may have contributed to the observed protection against ferroptosis in their model systems [10]. However, antioxidant properties were previously only detected at higher micromolar concentrations (up to 100 µM) of TRO, which may then contribute to protection against ferroptosis. To ensure that the protective effects observed here were achieved by the antioxidant effects of the applied compounds, we performed a DPPH assay with low protective concentrations (0.25 µM TRO and 25 µM ROSI), revealing no radical scavenging effects of the TZDs. Therefore, it can be assumed that the antioxidant properties of the TZDs were not involved in the protective effects against ferroptosis observed in the ACSL4/LPCAT2 OE cells in this study.

But the main question remains: how far are mitochondria involved in ACSL4/LPCAT-driven ferroptosis? The involvement of mitochondria in ferroptosis is still under debate. Mitochondrial involvement has been reported repeatedly in the literature, and various hypotheses have been discussed. In particular, lipid peroxidation at the membrane, cellular iron accumulation, and glutathione depletion have been described as the starting points of ferroptosis, which are then intensified in their severity by positive feedback loops [45]. Lipid peroxidation is, therefore, one of the starting points, but mitochondrial radical formation may also play a very decisive role, and these mechanisms are dependent on mitochondrial metabolism and (impaired) OXPHOS.

Some studies have suggested that mitochondrial dysfunction is a critical factor in the initiation of ferroptosis, while others have proposed that mitochondrial impairments play a secondary, downstream role in this process, representing another endpoint caused by ferroptosis. For instance, it was reported that the knockout of ACSL4 led to enhanced mitochondrial resistance to RSL3-triggered outer membrane rupture [10], supporting the claim of mitochondrial involvement in ACSL4-induced ferroptosis. On the other hand, it has also been reported that mitochondrial ROS levels in erastin-treated human cancer cells remain unchanged. Further, cells were still sensitive to ferroptosis after the elimination of mitochondria through mitophagy, suggesting that these organelles were not the main location where lipid peroxidation occurs during ferroptosis and their contribution to death mechanisms seemed to be dispensable [1,46,47].

In our previous studies in model systems for glutamate-mediated oxytosis in neuronal cells, we discovered that activation of the pro-apoptotic BCL2 family protein BH3-interacting-domain death agonist (BID) plays a crucial role in mitochondrial pathways of ferroptosis. Mitochondrial transactivation of BID led to severe disturbances in mitochondrial function and integrity [21,25,29,48,49]. These mitochondrial impairments ultimately led to the discharge and translocation of AIF from the mitochondria to the nucleus, resulting in caspase-independent, AIF-mediated cell death [17,29,48,50,51]. More recently, these findings were also confirmed in models of ferroptosis induced by erastin or RSL3 using both genetic approaches of BID knockout and pharmacological BID inhibitors to rescue neuronal cells from mitochondrial demise and ferroptotic cell death [25,52].

Further, dynamin-related protein 1 (Drp1) plays a pivotal role in regulating mitochondrial fission, and inhibiting Drp1 has beneficial effects on ferroptosis [49,53]. However, the role of Drp1 was not the primary focus of this study, as our findings suggest that mitochondria do not play a crucial role in the ACSL4/LPCAT2 model system beyond radical scavenging. Due to the overexpression of ACSL4/LPCAT2 in our model system, any further stabilization by Drp1 does not appear to be necessary. Nevertheless, recent evidence has linked Drp1 to ACSL4-driven ferroptosis since the Hsp90-dependent Drp1 dephosphorylation at serine 637 (Ser637) stabilizes and binds ACSL4, thereby increasing its expression in glioma cells and regulating ferroptosis through the generation of lipid ROS and alterations in mitochondrial morphology [54].

Here, we found that mitochondria play a critical role in ACSL4/LPCAT2-guided ferroptosis, represented by the alterations observed in mitochondrial parameters. Most importantly, mitochondrial ROS accumulation, loss of mitochondrial membrane potential, and reduced mitochondrial respiration were observed in the OE cells after exposure to RSL3, indicating a pivotal contribution of mitochondria in ACSL4/LPCAT2-driven ferroptosis in the HEK293T cells. Based on these data, we wanted to explore the particular importance of metabolic effects and mitochondrial ROS production in this paradigm of ferroptosis. In general, glutaminolysis is a metabolic pathway that converts glutamine into alpha-ketoglutarate, which is an important substrate for the tricarboxylic acid (TCA) cycle. The TCA cycle generates NADH and FADH2, which are required redox compounds during OXPHOS in the mitochondria. OXPHOS is the process by which ATP is generated through the transfer of electrons along the electron transport chain (ETC) in the mitochondrial inner membrane, coupled to proton pumping across the membrane. However, OXPHOS is also a major source of ROS, which are highly reactive and can damage cellular components. The shutdown of the TCA cycle and the inhibition of OXPHOS decreases the production of NADH and FADH2, which are the primary electron donors for the ETC. This reduces the electron flow along the ETC, resulting in a decreased generation of the proton gradient across the mitochondrial inner membrane. As a consequence, less ROS is produced due to a reduction in the leakage of electrons from the ETC to oxygen, which normally generates superoxide anions and other ROS. A decrease in ROS production can also be attributed to mild uncoupling, which refers to a physiological process where the coupling between the ETC and ATP synthesis is partially disrupted. This process can be achieved through the activation of uncoupling proteins (UCPs), among other mechanisms. When UCPs are activated, protons are allowed to re-enter the mitochondrial matrix without driving ATP synthesis. Consequently, the electron flow along the ETC is reduced, leading to a decrease in the generation of the proton gradient across the mitochondrial inner membrane. As a result, less ROS is produced due to a reduction in the leakage of electrons from the ETC to oxygen, which normally generates superoxide anions and other ROS. In ferroptosis, glutaminolysis plays a critical role in providing the necessary substrates for the TCA cycle. A recent study further identified the amino acid L-glutamine as a ferroptosis inducer, thereby fueling the cellular metabolic pathway glutaminolysis [44]. They demonstrated that glutamine was required for the initiation of ferroptosis and, therefore, the inhibition of glutaminolysis reduced ischemia/reperfusion-induced heart damage. However, in our system of HEK293T cells, glutamine deprivation failed to protect from ferroptosis, which is triggered by the overexpression of ACSL4 and LPCAT2 to such an extent that mitochondria and glutaminolysis may no longer play a critical role and are thus dispensable for further PUFA synthesis. In contrast, we found that mitochondrial function and integrity, which were massively impaired by RSL3 in the ACSL4/LPCAT2 overexpressing cells, was preserved by the mitochondria-targeted ROS scavenger MitoQ. Previous work in our lab showed that MitoQ operates as a mitochondria-targeted antioxidant, thereby preserving mitochondrial integrity and function in neuronal HT22 cells. Further, metabolic effects were observed in these neuronal cells in addition to mitochondrial ROS scavenging activities of MitoQ [8]. We, therefore, explored whether metabolic intervention alone may also rescue HEK293T OE cells from ferroptosis. Nevertheless, the attempt of metabolic intervention with the complex I inhibitor metformin in HEK293T cells further reduced mitochondrial respiration but did not prevent ferroptosis. The same was observed with the membrane penetrating complex I inhibitor phenformin and the complex II inhibitors itaconate and 4-octyl-itaconate. Thus, the metabolic effects of MitoQ were insignificant, while the antioxidant capacity mediated the observed protective effects at the level of mitochondria and at the cellular level. Conversely, protective effects of metformin were observed in erastin-treated neuronal HT22 cells, which substantiates the differences between the cell lines and between erastin and RSL3-induced ferroptosis. The susceptibility of HT22 cells to glutamate or erastin-induced ferroptosis depends on metabolic processes and (impaired) OXPHOS, and metabolic interventions inhibiting OXPHOS and driving glycolysis mediate protective effects in neuronal cells but not in HEK293T cells. However, the precise point at which metabolic switches or mitochondrial ROS production becomes the determining factor for ferroptosis mechanisms remains unknown. In contrast, when comparing HEK293T cells, another substantial discrepancy emerges, as these cells fail to trigger ferroptosis when exposed to erastin or glutamate or need notably higher concentrations of these ferroptosis inducers for induction of cell death. Another limitation of the study is the fulminant trigger of ferroptosis through lipid peroxidation via overexpression, which may exceed the potential involvement of mitochondrial mechanisms. Further, due to the stable transfection of the HEK293T cells with GFP-tagged constructs, the measurement of lipid peroxidation using the fluorescence probe BODIPY is not feasible. This limitation arises from the interference of GFP fluorescence with the detection of BODIPY signals, precluding accurate assessments of lipid peroxidation in this experimental setup.

Altogether, our data highlight the involvement of mitochondrial impairment in ferroptosis since mitochondrial ROS formation determined ACSL4/LPCAT2-driven oxidative death. Mitochondrial ROS scavenging by MitoQ protected HEK293T OE cells from oxidative damage, and these effects were independent of metabolic inhibition of mitochondria. In fact, metabolic intervention or glutamine starvation alone failed to prevent ferroptosis in the HEK293T cells, demonstrating the importance of mitochondrial ROS accumulation over metabolic mechanisms in paradigms of ACSL4/LPCAT2 overexpression. Mitochondrial mechanisms in cell death pathways of ferroptosis may be of use for future therapeutic approaches to various diseases. Overall, this study indicates that the role of mitochondria in ACSL4/LPCAT2-driven ferroptosis may vary depending on the cell type and context, emphasizing the need for further research to comprehensively grasp the mechanisms involved and the specific involvement of mitochondria in oxidative cell death.

## Data Availability

The data that support the findings of this study are available within the article and its Supplementary Materials.

## Supplementary Materials

The following supporting information can be downloaded at https://www.mdpi.com/article/10.3390/antiox12081590/s1. Figure S1: Protein levels of system Xc^−^ in LV and OE cells; Figure S2: Real-time measurement of LV and OE cells after RSL3 treatment and the effects on GSH levels; Figure S3: Trolox and Fer-1 prevent ACSL4/LPCAT2-driven ferroptosis; Figure S4: Protective effects of MitoQ in HEK293T cells; Figure S5: Complex I inhibition by phenformin failed to prevent ferroptosis; Figure S6: DPPH scavenging effect of the 5-LOX inhibitors zileuton and ST1853.
