# Supplementary material for: Mitochondrial Reactive Oxygen Species Formation Determines ACSL4/LPCAT2-Mediated Ferroptosis
Source: Antioxidants (Basel). 2023 Aug 9;12(8):1590. doi: 10.3390/antiox12081590 (PMC10451816; doi:10.3390/antiox12081590)
Supplement: Supplementary file 1 [file antioxidants-12-01590-s001.zip › antioxidants-2548153-supplementary.pdf]

Supplemental Figure S1

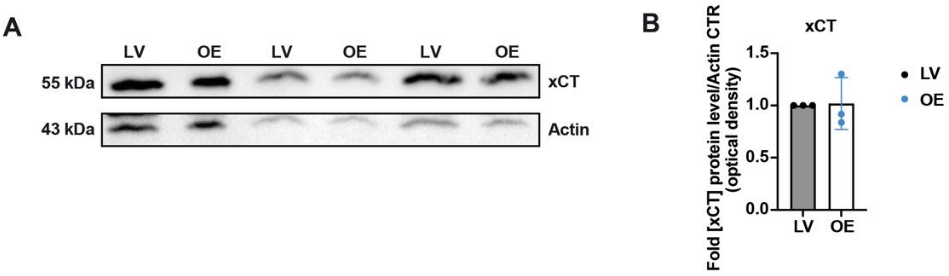

Figure S1. Protein levels of system Xc⁻ in LV and OE cells.

A Western Blot analysis (n = 3 replicates) of the protein xCT in LV and OE cells. The protein bands were quantified with reference to the loading control actin. **B** Respective quantification of the protein levels for LV and OE cells. Not declared: not significant (ANOVA, Scheffé's test).

Supplemental Figure S2

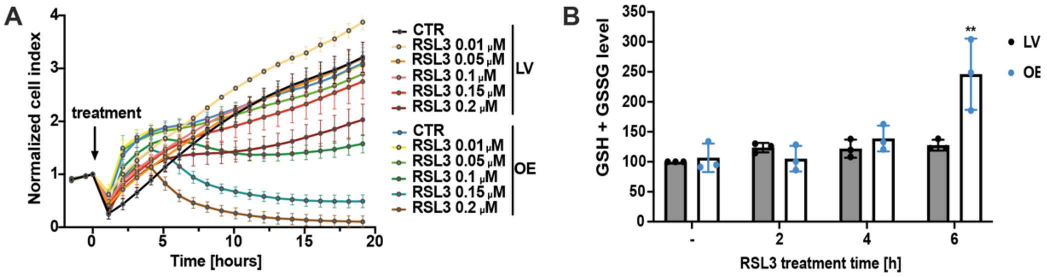

Figure S2. Real time measurement of LV and OE cells after RSL3 treatment and the effects on GSH levels.

A Representative real-time impedance measurements for the evaluated treatment time of 19 hours depicted in Fig. 1 H). **B** GSH+GSSG levels are provided as percentage of the respective control conditions for different RSL3 treatment time points (n = 3 replicates). ** P < 0.01 compared to control condition (ANOVA, Scheffé's test).

Supplemental Figure S3

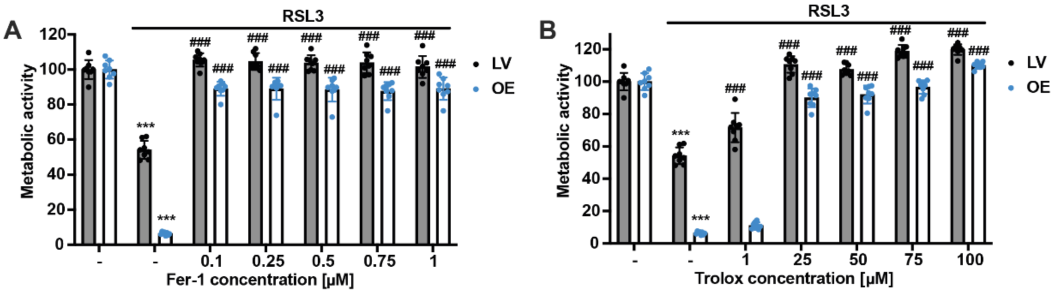

Figure S3. Trolox and Fer-1 prevent ACSL4/LPCAT2-driven ferroptosis.

A Metabolic activity was determined by MTT assay after 16 hours of co-treatment with 0.1 µM RSL3 and 0.1-1 µM ferrostatin-1 or **B** 1-100 µM trolox in LV and ACSL4/LPCAT2 OE cells. *** P < 0.001 compared to (treated) control condition; ### P < 0.001 compared to RSL3-treated control cells (ANOVA, Scheffé's test).

Supplemental Figure S4

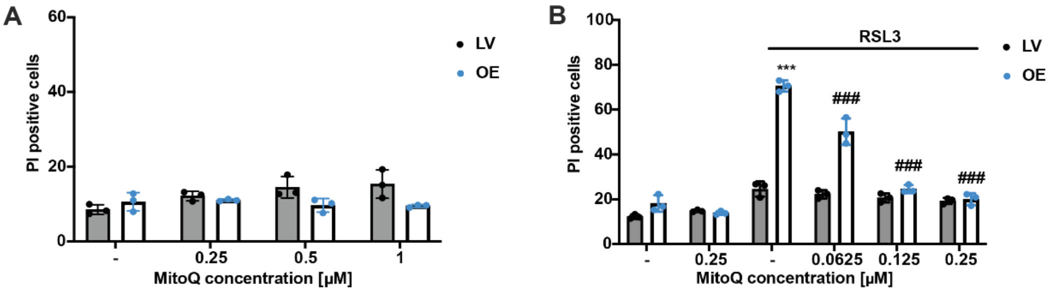

Figure S4. Protective, but no toxic effects of MitoQ in HEK293T cells.

Cell death was quantified by flow cytometry and PI staining **A** after 6 hours of 1 µM RSL3 treatment and co-treatment with different MitoQ concentrations (0.0625-0.25 µM) **B** after 16 hours treatment time of different MitoQ concentrations (0.25-1 µM) (5 000 cells per replicate of n = 3 replicates, percentage of gated cells). *** P < 0.001 compared to control condition, ### P < 0.001 compared to RSL3-treated condition (ANOVA, Scheffé's test).

Supplemental Figure S5

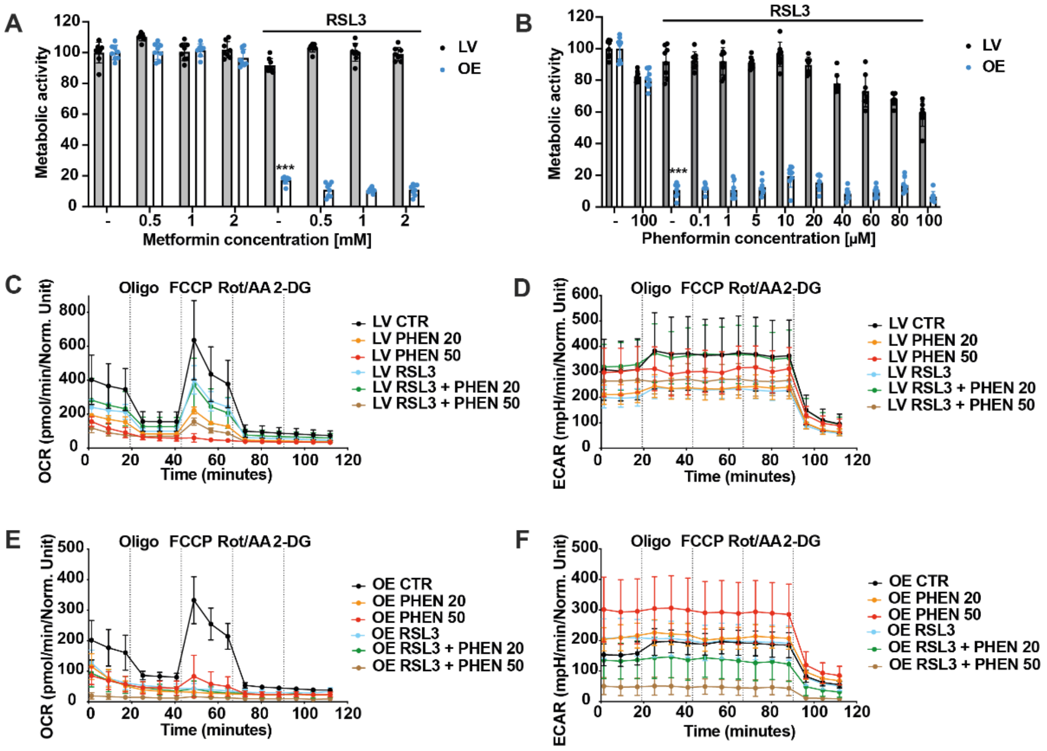

Figure S5. Complex I inhibition by phenformin failed to prevent ferroptosis.

A Metabolic activity was determined by MTT assay after 22 hours of co-treatment with 0.1 µM RSL3 and 0.5-2 mM metformin in both cell lines. **B**

Metabolic activity was determined by MTT assay after 22 hours co-treatment with 0.1 μM RSL3 and 0.1-100 μM phenformin in LV and ACSL4/LPCAT2 OE cells. **C, E** Mitochondrial respiration and **D, F** glycolysis were measured in LV (C, D) and ACSL4/LPCAT2 OE (E, F) cells after 16 hours treatment with 1 μM RSL3 and 20 μM or 50 μM phenformin (PHEN). Data are shown as percentage of control conditions with n = 8 replicates per condition). *** P < 0.001 compared to control conditions (ANOVA, Scheffé's test).

Supplemental Figure S6

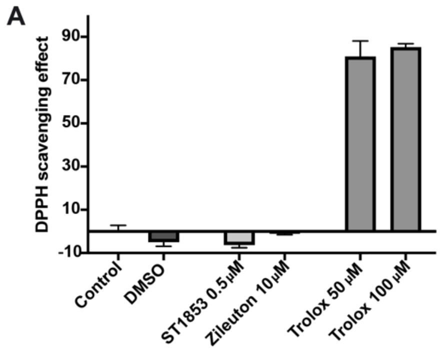

Figure S6. DPPH scavenging effect of the 5-LOX inhibitors zileuton and ST1853.

A For the determination of antioxidant properties of the substances, an DPPH assay was performed with 50 μM and 100 μM trolox, 0.5 μM ST1853 and 10 μM zileuton. Data are given as mean ± SD (n = 6-8 replicates).
